# Recent advances in the multifaceted mechanisms of catalpol in treating osteoporosis

**DOI:** 10.3389/fphar.2025.1560715

**Published:** 2025-03-04

**Authors:** Na Li, Xiaoying Mu, Shudong Zhang, Huaxin Wang

**Affiliations:** ^1^ Shandong Co-Innovation Center of Classic Traditional Chinese Medicine Formula, Shandong University of Traditional Chinese Medicine, Jinan, China; ^2^ The Second Affiliated Hospital of Shandong University of Traditional Chinese Medicine, Shandong University of Traditional Chinese Medicine, Jinan, China; ^3^ Binzhou People’s Hospital, Binzhou, China

**Keywords:** catalpol, osteoporosis, pharmacological mechanisms, osteogenesis, osteoclasts

## Abstract

Catalpol (CAT) is a landmark active ingredient in traditional Chinese medicine Rehmannia (TCT), also known as dehydroxybenzoate catalpone, which is a kind of iridoid terpene glycoside with strong antioxidant, anti-inflammatory, antitumor and other biological activities. It can exert its anti-disease effect in a variety of ways. For some patients with chronic diseases, the application of azalea alcohol in rehmannia may bring more comprehensive and long-lasting efficacy. Studies have shown that the anti-disease effect of catalpol in osteoporosis (OP) is mainly achieved through various pathways such as Wnt/β-catenin signaling pathways to promote osteogenic differentiation, and RANKL/RANK and other signaling pathways to inhibit osteoclastic differentiation. At present, there is a slight lack of analysis of the mechanism of action of catalpa alcohol in the treatment of osteoporosis, so this study comprehensively searched the literature on the mechanism of action of catalpa alcohol in the treatment of osteoporosis in various databases, and reviewed the research progress of its role and mechanism, to provide reference and theoretical basis for the further development and application of catalpol.

## 1 Introduction

Osteoporosis (OP) represents a prevalent systemic skeletal condition marked by diminished bone density and compromised microstructural integrity. This deterioration results in weakened osseous tissue, heightened susceptibility to fractures, and reduced bone resilience (Silverstein et al., 2024). Etiologically, osteoporosis can be classified into two main categories: primary and secondary forms (Ayers et al., 2023). Evidence from medical literature points to osteoporosis affecting close to 30% of people who have surpassed their fifth decade of life, affecting over 200 million people worldwide (The Lancet Diabetes Endocrinology, 2021). Therefore, exploring novel preventive and therapeutic approaches is imperative.

In traditional Chinese medicine (TCM), osteoporosis falls under the categories of “bone flaccidity” and “bone impediment,” with the pathogenesis linked to a deficiency of essence leading to inadequate nourishment of the bones. Historically, in the research of osteoporosis treatment, Chinese herbal medicine has shown distinct advantages due to its significant efficacy, minimal toxic side effects, and suitability for long-term use (Duan et al., 2023). Common anti-osteoporosis herbal prescriptions often emphasize kidney-nourishing herbs including Rehmannia glutinosa, Epimedium, Drynaria, Cornus officinalis, and Astragalus (Jinlong et al., 2020; Zhang et al., 2021). These are known to mitigate the reduction in trabecular bone mineral density (BMD), increase cortical bone thickness, and enhance trabecular bone formation in the bone marrow space. They also promote osteoblast proliferation, activity of alkaline phosphatase (ALP), and expression of osteoprotegerin (OPG), while reducing the number of TRAP-positive multinucleated cells and osteoclasts (Lim and Kim, 2013). Contemporary studies have elucidated the mechanisms and efficacy of TCM’s bioactive compounds in combating osteoporosis, with the majority involving various glycosides, saccharides, organic acids, amino acids, and inorganic elements (Carnovali et al., 2018). Among these, catalpol has been identified as one of the most active components (Wang, 2015; Xia, 2019; Liu et al., 2017), which is listed as a marker compound in the content determination of Rehmannia glutinosa in the *Pharmacopoeia of the People’s Republic of China* (2020 edition).

**FIGURE 1:**
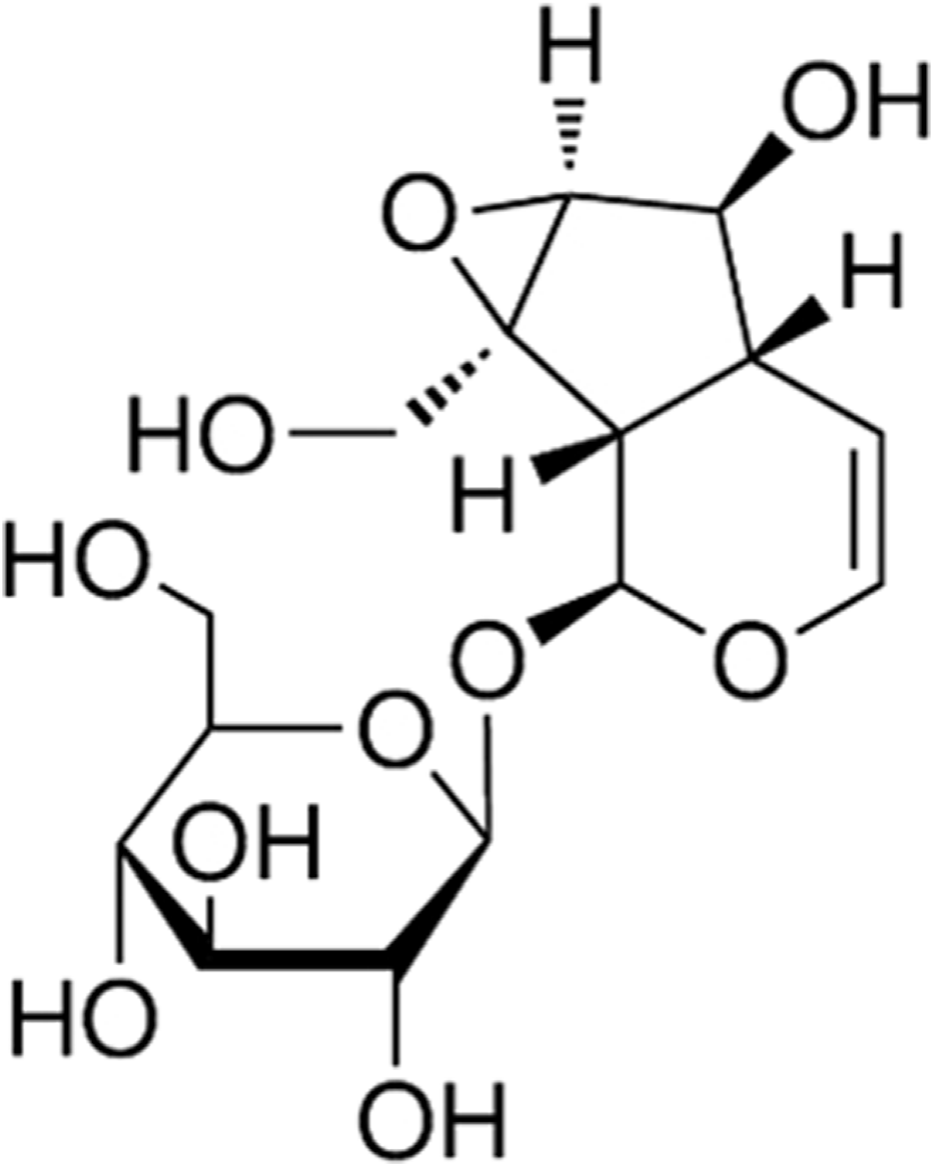
The chemical structural formula of CAT (C_15_H_22_O_10_).

Catalpol is a type of flavanone glycoside. Studies have revealed its capabilities in lowering blood sugar (Shieh et al., 2011), anti-fibrotic effects on the kidneys (Sun et al., 2020), neuroprotection, and combating cardiovascular and cerebrovascular diseases (Hongwei and Meng, 2015) (for the chemical structure of catalpol, see Figure 1). Consequently, it has attracted widespread attention from researchers and clinicians. Researchers and doctors have studied the anti-osteoporotic effects of catalpol at the molecular mechanism level, providing theoretical foundations and support for its clinical applications. It has been discovered that catalpol promotes osteogenic differentiation and inhibits osteoclast differentiation through various molecular mechanisms, thereby achieving therapeutic effects (Table 1). While the anti-osteoporotic effects of catalpol have been confirmed by numerous studies, there is a relative scarcity of comprehensive reviews on its mechanisms against osteoporosis. Based on this, the current article reviews the molecular mechanisms of catalpol in combating osteoporosis, aiming to provide theoretical bases and references for researchers and clinicians and to suggest new avenues for the further development and utilization of catalpol.

**TABLE 1:** Anti-OP effects of CAT and its mechanisms.

Real modules	Effective concentrations	Possible mechanisms	Reference
BMSCs	10, 50, 250 μM	Activates the Winter/β-Catenin signaling pathway	Zhu et al. (2019)
	0, 10, 25, 50, 100, 250, 500 μM	The JAK 2/STAT3 signaling pathway promotes osteogenesis, angiogenesis and their coupling	Chen et al. (2021a)
	0.05, 0.1, 0.2, 0.5 mg/L 1,2,5,10 mg/L; 20,50,100 mg/L	0.05∼10 mg/L could promote proliferation, and the proliferative effect was the strongest at 1.0 mg/L, and there was no obvious proliferative effect when the concentration was greater than or equal to 20 mg/L 0.2∼100 mg/L could increase ALP activity, and 2.0 mg/L had the strongest ALP promotion ability. All concentrations increased the number of mineralized nodules and promoted the secretion of calcification-related proteins	Fu and Zhang (2012)
	1 × 10^−3^,1 × 10^−5^ 1 × 10^−7^,1 × 10^−9^ mol/L	At 48 h, the activities of 1 × 10^-7^ mol/L and 1 × 10^-3^ mol/L ALP increased, and the promotion effect of 1 × 10^-3^ mol/L was more significant.1 × 10^-5^ mol/L and 1 × 10^-3^ mol/L BGP activity increased. At 72 h, the activities of ALP and BGP in the 1 × 10^-3^ mol/L group were significantly higher than those in the others	Jia et al. (2015a) and Jia et al. (2015b)
	1.0 mg/L	It increased the mRNA expression of Wnt5a, Wnt11 and β-catenin, activated the canonical and non-canonical Wnt/β-catenin signaling pathways, promoted the expression of RUNX2 and OCN, and promoted the proliferation of BMSCs	Fu et al. (2014)
	1 × 10-5 mol/L	It increased the expression of Smad4 mRNA and activated the Wnt/β-catenin signaling pathway	Jia et al. (2015a)
	1 × 10^−3^,1 × 10^−4^ 1 × 10^−5^,1 × 10^−6^ 1 × 10^−7^,1 × 10^−8^ 1 × 10^-9^ mol/L	Upregulated the expressions of Shh, Ptch1, Smo and Gli1, and activated the Hedgehog signaling pathway	Stefanaki et al. (2018)
	10 mg/L	Increases osteoblast-induced expression of osteoprotein and RANKL	He et al. (2020)
	10, 40, 160 mM	It increased the expression of PKD1 and activated the PKD 1/Sirt 1 pathway, increased the expression of ALP, RUNX2 and BMP9, and promoted proliferation and osteogenic differentiation	Veis and O'Brien (2023)
	10, 40, 160 mM	Activates the PKD1/Sirt1 pathway	Xu et al. (2023)
	10, 30 mg/kg	It enhances bone mineral density, significantly enhances the bone healing ability of MSCs in defects, and promotes the differentiation of BMSCs into mature osteoblasts *in vitro*. Enhance osteogenic activity and promote bone regeneration	Wu et al. (2024)
	0, 50, 100 μg/mL	It reduces the expression of M1-related factors, promotes osteogenesis and angiogenesis, and inhibits osteoclast production	Wu et al. (2022)
MC3T3-E1	1 × 10^−5^, 1 × 10^−6^ 1 × 10^−7^, 1 × 10^−8^ 1 × 10^-9^ mol/L	1 × 10^−7^∼1 × 10^-9^ mol/L promoted value-added, 1 × 10^−5^∼1 × 10^-9^ mol/L culture enhanced ALP activity for 48 and 72 h, BGP activity increased after 8 and 12 days of culture, and the number of mineralized nodules increased after 19 days of culture	Chen et al. (2021a)
	1, 10, 100, 500, 800, 1000, 2000, 4000 mg/L	>500 mg·L-1 significantly promotes the secretion of ALP; The number of calcium nodules in the extracellular matrix increased, and the expression of Runx2, Bglap and Col1a1 was promoted	Luyi (2021)
		The expression of IGF-1R and P-IGF-1R was increased, and the expression of GSK-3β and PPAR-γ decreased	Zhang (2016)
	1, 10 μM	Promote the proliferation and differentiation of MC3T3-E1 under high glucose damage conditions	Zhao et al. (2021)
		Improve cell proliferation and ALP activity, express BMP2, Runx 2, Osterix and p-Smad 1/5/9, and activate PI3K/Akt/mTOR signaling pathway	Gong et al. (2019)
	0.01–1 μM	Inhibits the weakening of cellular activity, inhibits increased apoptosis and autophagy, inhibits cytochrome P450 1A1 and extracellular signal-regulated kinase levels; Superoxide dismutase and extracellular signal-regulated kinase 1 gene expression were repaired, and glutathione peroxidase 4 and ALP and osterix were significantly increased	Choi et al. (2019)
	12.25, 24.5, 49 μg/ml	The expression of Bcl-2 and Bax was increased, the expression of Caspase-3 was decreased, and the survival rate and activity of cells after OGD treatment were improved	Ju et al. (2018)
Osteoclasts	0.05, 0.1, 0.5, 1 mg/L; 2, 5, 10 mg/L; 20, 50, 100 mg/L	It stimulates the proliferation of OB, increases the expression of ALP and Erβ, and inhibits OC activity	Lai et al. (2015a)
	0.05, 0.5, 5, 50, 100 mg/L	0.05 mg/L significantly increased the expression of OPG, blocked the overexpression of OPGL, inhibited OC bone resorption, and promoted bone formation	Lai et al. (2016)
	5, 10, 20 mg/kg	The expressions of Sirt 6, ERα, FasL, cleaved-caspase 8, cleaved-caspase 3 and Bax were upregulated, and the expressions of NFATc 1, Ctsk, Oscar and Trap were downregulated, and the apoptosis of osteoclasts was promoted through the Sirt 6-ERα-FasL axis	Chen et al. (2024)
	0, 100, 200, 400 µM	Inhibition of RANKL induces osteoclast production and activity by inhibiting the PTEN/NF-κB/AKT signaling pathway	Meng et al. (2020)
	0, 6.25, 12.5, 25, 50, 100, 200, 400, 800 uM	Increasing PTEN expression decreased NFATc1, c-Fos, CSTK and TRAP expression by inhibiting the activation of NF-κB and AKT signaling pathways in RNAKL/RANK signaling	Meng (2020)
SD rats	50 mg/kg	Upregulation of BMP-2 expression activates the increase of Wnt/β-catenin signaling pathway	Cao et al. (2021)
	0.06,1,4 g/kg	The activities of ALP and OCN were significantly increased, the content of TRAP was decreased, and the femoral bone mineral content, bone mineral content, bone tissue mineral density, bone tissue mineral content, bone volume fraction, trabecular bone thickness, and trabecular bone separation were all increased	Zhang (2016)
	20,100 mg/kg	It improves the damage of bone density, bone microstructure, bone morphology and bone mass, reduces the expression of ALP, COL1, Runx2 and BMP9, and induces GSK3b phosphorylation. Reverses the decrease in serum GSH and SOD and the increase in serum MDA	Veis and O'Brien (2023)
	0.2 mL/kg	reduces the expression of calcium-binding protein S100A12, interleukin 1β and galectin 3 inflammatory factors	Zhang et al. (2020)
	30, 90 mg/kg	improves OB activity	Zhao et al. (2021)
	2 μg/kg	Inhibits TNF-α and COX-2 expression, alleviates alveolar bone damage and loss, and promotes alveolar bone mineralization	Li et al. (2017b)
	10, 20, 40 mg/kg	Expression of major transcription factors that promote the differentiation of Th 2 cells and inhibit the expression of Th 1 cells	McWhorter et al. (2013)

## 2 Catalpol promotes osteoblastic differentiation

**FIGURE 2:**
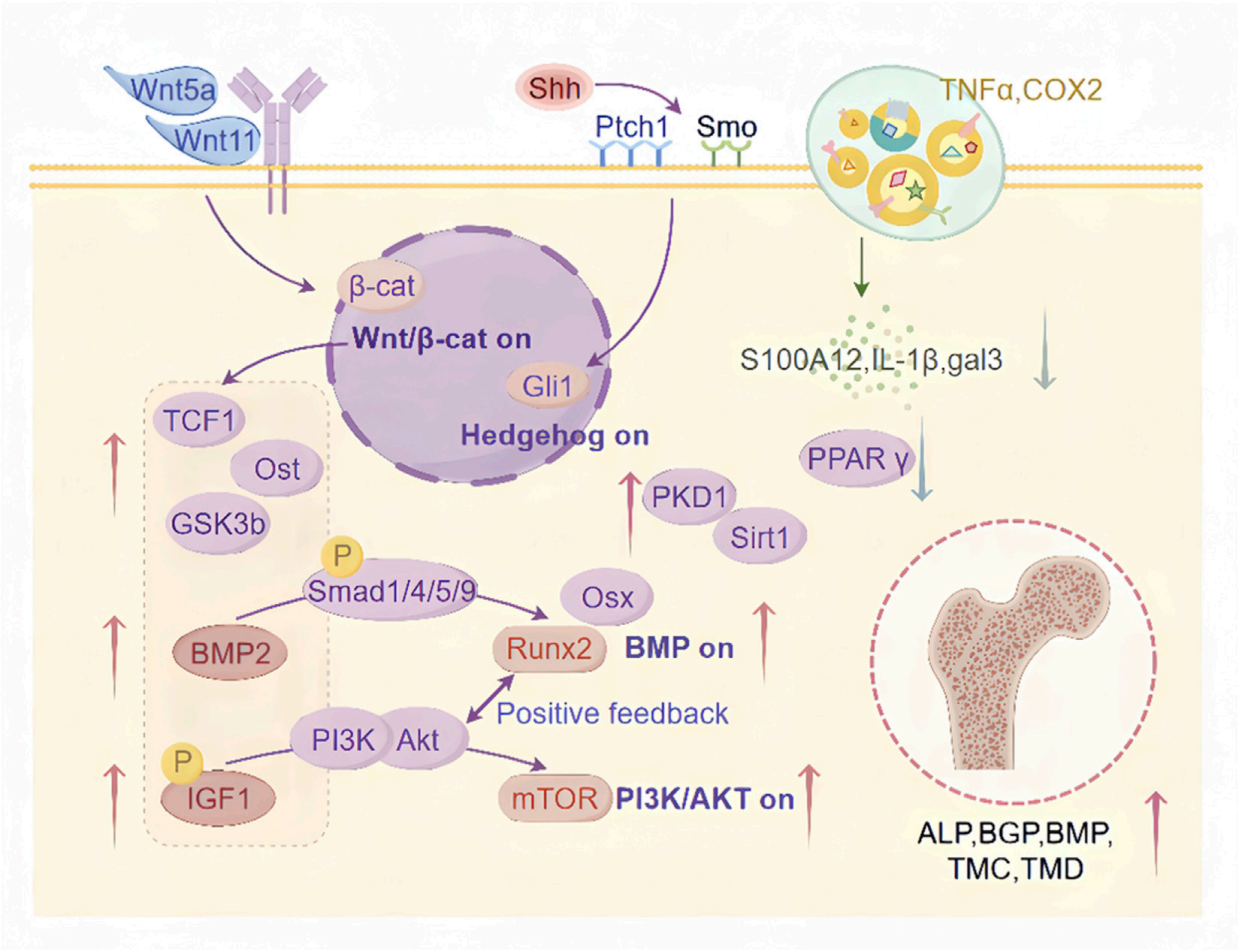
Mechanisms by which CAT promotes osteoblast differentiation. This includes the regulatory roles of CAT in activating the Wnt/β-catenin signaling pathway, Hedgehog signaling pathway, inflammatory signaling pathways, BMP signaling pathway, IGF-1/PI3K/Akt/mTOR signaling pathway, PKD1/Sirt1 signaling pathway, and their downstream targets, promoting osteogenic differentiation and inhibiting adipogenic differentiation through multiple regulatory mechanisms.

The equilibrium of mineral content in bones is sustained through the bone remodeling process, wherein osteoclasts (OCs) facilitate resorption while osteoblasts (OBs) promote formation and the integrity of bone structure (Langdahl, 2021). Abnormal development and differentiation of OBs and OCs, leading to a predominance of bone resorption over bone formation, constitute a critical pathogenic mechanism in OP. Enhancing osteoblastic differentiation is a key aspect of combating OP (Figure 2).

Research findings indicate that CAT administration results in an augmentation of trabecular quantity and reduce their degenerative structural changes, significantly ameliorating trabecular deterioration and reducing bone loss (Zhu et al., 2019), while also promoting bone regeneration and angiogenesis (Chen et al., 2021a). CAT enhances the activity of alkaline phosphatase (ALP), bone gamma-carboxyglutamate protein (BGP), and bone morphogenetic proteins (BMP) in mouse osteoblastic precursor cells MC3T3-E1 (Wu et al., 2010) and bone marrow mesenchymal stem cells (BMSCs) (Fu and Zhang, 2012). It increases the number of mineralized nodules and promotes the expression of osteogenesis-related genes and osteoblastic activity, all within a broad range of safe concentrations (Louie et al., 2020). The effects are most significant at CAT concentrations of 1 × 10^−3^ mol/L and 1 × 10^−5^ mol/L (Jia et al., 2015a), notably accelerating BMP formation and secretion in MC3T3-E1, enhancing alkaline phosphatase activity, and proliferating the count of mineralized nodules (Jia et al., 2015b; Yang, 2021). These findings suggest that CAT can prevent and mitigate the development of OP by modulating osteoblastic differentiation.

### 2.1 Wnt/β-catenin signaling pathway

Multiple physiological functions are intricately linked to the Wnt/β-catenin signaling route (Liu et al., 2022) and is also believed to be integral to osteoblastic differentiation (Zhu et al., 2019). CAT may stimulate Runx2 and multiple targets related to bone formation by upregulating the Wnt/β-catenin transcription factor 1 (TCF1) signaling regulatory element located in the promoter of the Runx2 gene, such as increasing the expression of os-teocalcin (ost), one of the main indicators for osteoblast differentiation and maturation into mineralization, increasing the secretory deposition of ALP, promoting the differentiation of BMSCs in the direction of osteoblasts and the maturation of newly formed osteocytes (Gaur et al., 2005). This effect is related to the simultaneous activation of both the classical and non-classical Wnt signaling pathways (Fu et al., 2014; Fu and Zhang, 2012). Activated β-catenin stimulates the formation of osteoblasts via the Wnt/β-catenin pathway, inducing an increase in ALP activity (Bennett et al., 2005; Jackson et al., 2005). CAT has been reported to promote the accumulation of intracellular β-catenin by inducing Wnt5a and Wnt11, and the accumulated β-catenin is transported to the nucleus, forming a complex with the transcription factor LEF/TCF, stimulating downstream gene expression (Huang et al., 2019), activating classical Wnt signaling, and thereby promoting the proliferation of BMSCs (Fu et al., 2014). It also activates multiple pathways in the Wnt/β-catenin signaling network by upregulating key regulatory proteins such as BMP-2 (Cao et al., 2021), Smad4 (Jia et al., 2015b), and downregulating GSK3b, as well as increasing the expression of IGF-1R and P-IGF-1R (Zhang, 2016), thus promoting osteoblastic differentiation.

### 2.2 Hedgehog signaling pathway

A fundamental role in embryo development, cellular proliferation, differentiation, and tissue/organ genesis is played by the evolutionarily conserved Hedgehog pathway (Chen et al., 2023). CAT can directly bind to the membrane receptor Ptch1 by upregulating the upstream ligand Shh signaling molecule in the Hedgehog signaling pathway, which relieves the inhibitory effect of Ptch1 on Smo, transmits Hedgehog signaling to the cytoplasm, activates the expression of downstream nuclear transcription factor Gli1, and promotes the differentiation of BMSCs into osteogenic (Lai et al., 2019). Exploring the mechanisms of this pathway facilitates the identification of specific targets for the effect of tsozinol on BMSCsin combating and managing OP.

### 2.3 Inflammatory signaling pathways

Inflammatory responses exacerbate the prevalence of OP by inhibiting bone formation, promoting bone resorption, and suppressing the proliferation and differentiation of myocytes (Stefanaki et al., 2018). CAT exhibits significant anti-inflammatory and antioxidant effects, inhibiting the expression of inflammatory mediators TNF-α and the key enzyme COX-2 involved in prostaglandin synthesis at mRNA and protein levels, thereby restoring the secretion levels of ALP and OCN (Liu et al., 2016). It increases the expression of osteoprotective factors and nuclear factor κB receptor activator ligand, altering the microstructure of bone through abnormal bone remodeling and reduction of mineral content (He et al., 2020). Reducing the expression of calcium-binding protein S100A12, interleukin 1β (IL-1β), and galectin 3 (gal-3) inflammatory factors plays an important role in the progression of early bone disease (Zhang et al., 2020).

### 2.4 BMP signaling pathway

The Bone Morphogenetic Protein (BMP) regulated pathways are critical for promoting ossification. These cascades regulate osteoblastic differentiation marker expression (e.g., ALP, OPN, bone sialoprotein, OCN), suppressing adipogenesis while promoting osteogenesis through Runx2 and Osterix (Osx) modulation in osteoblasts (Cheng and Genever, 2010; Komori, 2005). There are known two subtypes of BMP receptors, BMPR-I and BMPR-II, which are serine-threonine kinase receptors. Upon binding of BMP to BMPR-II, BMPR-I is recruited to form an activated quaternary complex, which then phosphorylates and activates the intracellular Smad protein. Receptor Smads bind to co-Smads and translocate into the nucleus as transcription factors. One of the BMP-Smad target genes is Runx2 (Lin and Hankenson, 2011). It is through this mechanism that CAT positively regulates the expression of BMP2, thereby significantly increasing the expression of Runx 2, Osx and p-Smad 1/5/9 in MC3T3-E1 cells, and participating in the physiological process of osteogenesis (Gong et al., 2019), indicating that CAT’s osteogenic effects may be attributed to its influence on BMP signaling cascades.

### 2.5 IGF-1/PI3K/Akt/mTOR signaling pathway

Diabetes-induced bone loss or osteoporosis, termed diabetic osteoporosis (DOP), exhibits impaired osseous repair and renewal, accompanied by elevated fracture susceptibility (Napoli et al., 2017). Evidence suggests CAT protects against glucose-mediated bone deterioration by optimizing osteoblast performance, fostering their expansion and specialization in hyperglycemic MC3T3-E1 environments, and minimizing hindrances to skeletal mineralization (Zhao et al., 2021). Insulin-like growth factor (IGF) is involved in blood glucose regulation and influences osteoblast proliferation and survival, thus regulating bone remodeling, with its production and storage in the bone matrix (Ghodsi et al., 2016; Zhang, 2016). CAT can bind correctly within the binding pocket of IGF-1, further indicating that IGF-1 is one of the targets of CAT. The binding strength of CAT with the IGF-1 binding pocket is strong, and it substantially modulates IGF-1’s expression and phosphorylation levels (Oh et al., 2003). CAT stimulates osteoblast proliferation and differentiation by activating the PI3K-Akt pathway, which in turn activate It also activates multiple pathways in the Wnt/β-catenin signaling network by upregulating key regulatory proteins such as BMP-2 (Cao et al., 2021), Smad4 (Jia et al., 2015), and downregulating GSK3b, as well as increasing the expression of IGF-1R and P-IGF-1R (Zhang, 2016), thus promoting osteoblastic differentiation.

p-IGF-1, p-PI3K, and p-mTOR (Gong et al., 2019; Bakker and Jaspers, 2015; Bakker et al., 2016). Runx2 and the PI3K/Akt/mTOR pathway are interdependent in the regulation of osteoblast differentiation. Runx2 upregulates the protein levels of PI3K subunits and Akt, while the PI3K/Akt/mTOR pathway significantly enhances Runx2’s DNA-binding and Runx2-dependent transcription (Sheng et al., 2014). This positive feedback loop is also one of the ways CAT significantly promotes osteoblast differentiation and migration, suggesting that CAT in the prevention and treatment of DOP can augment both skeletal mineral accretion and collagen synthesis.

CAT can also activate the estrogen signaling pathway to affect the interaction between downstream transcription factors and the PI3K/AKT signaling pathway. Activation of the PI3K/AKT signaling pathway leads to the upregulation of endothelial eNOS and an increase in NO levels, which stimulates angiogenesis, enhances the local blood supply to bone tissue, and improves microcirculatory structure. Thus, these processes favor bone formation (Simão et al., 2012; Li et al., 2017b; Sun et al., 2019). Literature reports suggest that the interaction between ER and the PI3K/AKT signaling pathway may depend on the binding of scaffold proteins and adaptor proteins to the p85 subunit of PI3K to form adherent plaque protein complexes and ERα, which activate downstream AKT or AKT 2, thereby triggering cascades in the signaling pathway (Sun et al., 2001). When used, activated AKT phosphorylates serine residues in the AF-1 region of ERα, thereby regulating the effect of IGF-1 on Erα (Martin et al., 2000).

### 2.6 PKD1/Sirt1 signaling pathway

Among secondary osteoporosis cases, glucocorticoid-induced osteoporosis (GIOP) stands as the most prevalent form. Elevated oxidative stress impairs the osteogenic potential of murine preosteoblasts and compromises their skeletal structure, but antioxidants can reverse this process (Atashi et al., 2015). As an antioxidant (Jiang et al., 2004), CAT upregulated PKD1-related pathways by increasing the activity of the promoter of the mechanoductive molecule polycystic kidney disease-1 (PKD1), promoted the expression of Sirt1 with strong antioxidant activity, and increased the expression of ALP activity and RUNX 2 in BMSCs under oxidative stress (Zhang et al., 2017), and activation of SIRT 1 can promote angiogenesis and osteogenic differentiation in BMP 9-induced MSCs (Lu et al., 2022). Bone marrow PKD1/Sirt1 signaling is enhanced by CAT in murine models of GIOP. Similarly, in BMSCs treated with dexamethasone (Dex), CAT also promotes their proliferation and osteogenic differentiation (Xu et al., 2023). Therefore, CAT holds promise as a safe and effective therapeutic for GIOP.

### 2.7 Inhibition of adipogenic and promotion of osteogenic differentiation

Peroxisome proliferator-activated receptor gamma (PPAR-γ) is a member of the ligand-activated nuclear transcription factor superfamily (Watt and Schlezinger, 2015). CAT can reduce the protein expression of PPAR-γ, suppressing BMSC adipogenesis while enhancing their osteoblastic lineage commitment (Zhang, 2016).

## 3 Inhibition of osteoclast differentiation

**FIGURE 3:**
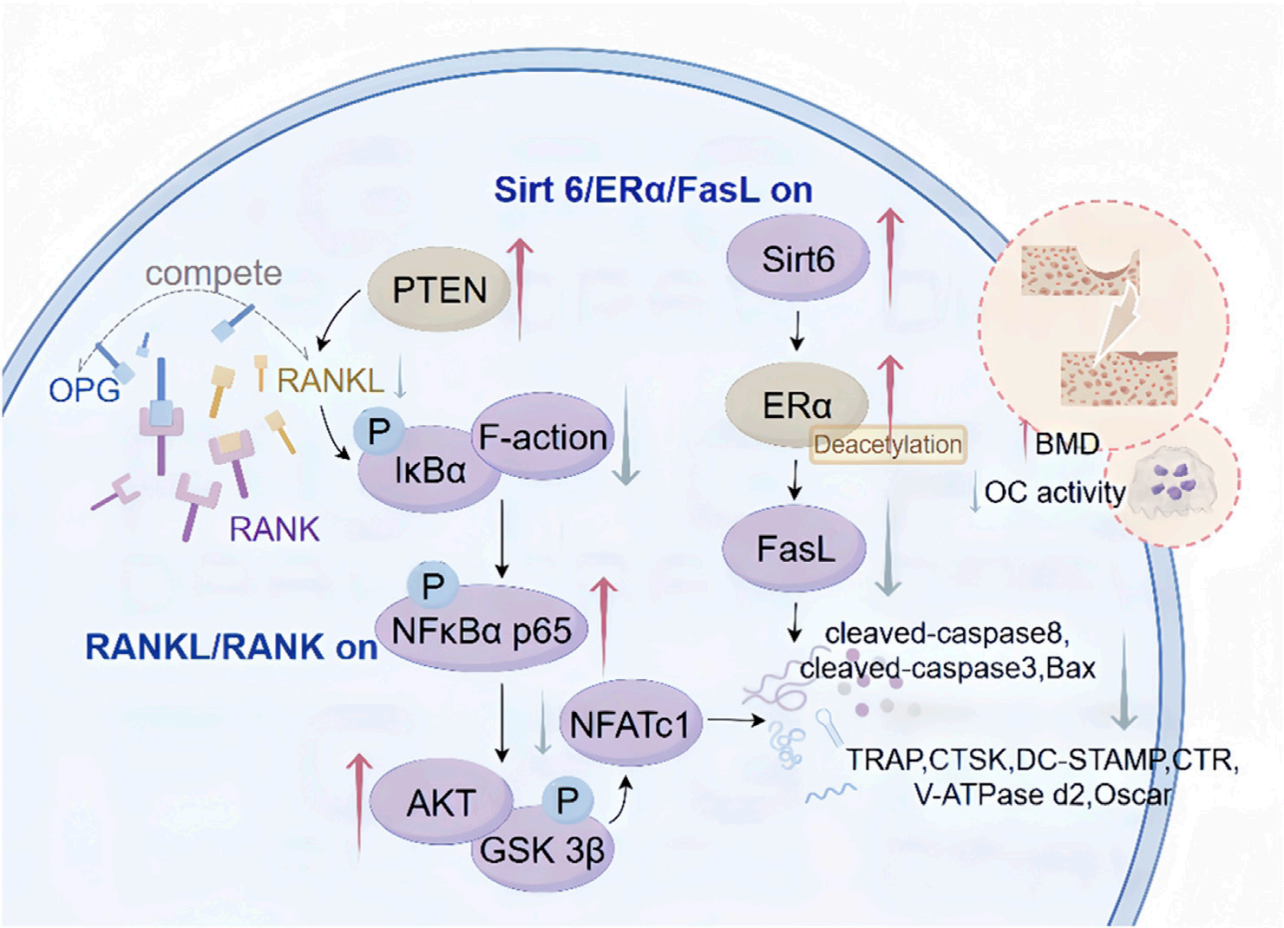
Mechanism of CAT inhibition of osteoclast differentiation. These include the regulatory mechanism between CAT initiating the RANKL/RANK signaling pathway through upstream signaling, the regulatory role between the Sirt 6/ERα/FasL signaling pathway and their downstream targets, inhibiting osteoclast differentiation, and intervening in the apoptosis process.

The foremost bone-eroding cells, known as osteoclasts, are descendants of the monocyte/macrophage hematopoietic lineage. They absorb bone to maintain a matrix with appropriate strength and elasticity to meet structural demands and assist in calcium homeostasis. Nevertheless, the overproduction or hyperactivation of osteoclasts may result in bone degradation. resulting in OP and other bone diseases (Veis and O'Brien, 2023). A more comprehensive understanding of osteoclast biology could offer more specific therapeutic directions for the diagnosis and treatment of OP (Figure 3).

CAT has been shown to significantly ameliorate the excessive formation of OCs (Sun et al., 2022). Lai et al. discovered that a concentration of 0.05 mg/L CAT significantly reduced the number of OC-mediated bone resorption pits after 48, 72, and 96 h in an OB-OC co-culture system, indicating that CAT can decrease OC activity. Furthermore, CAT inhibits the secretion of tartrate-resistant acid phosphatase (TRAP), a lysosomal enzyme involved in the degradation of bone matrix minerals such as calcium and phosphate released by OCs (Lai Manxiang et al., 2015; Lai et al., 2016; Chen et al., 2024). Consequently, The function of CAT includes suppressing osseous tissue degradation. thus providing bone protective effects and exerting anti-osteoporosis (OP) actions.

### 3.1 RANKL/RANK signaling pathway

The receptor activator of nuclear factor-kappa B ligand (RANKL) binds to the receptor activator of nuclear factor-kappa B (RANK) on the surface of OC precursor cells and OCs, triggering a cascade of reactions that lead to the differentiation, maturation, and activation of OCs. The RANKL/RANK signaling pathway is instrumental in orchestrating the delicate interplay between OCs and OBs, as well as in bone metabolism and remodeling, and has become a new target for the prevention and treatment of metabolic bone diseases (Yue et al., 2022). Osteoprotegerin (OPG) acts as a decoy receptor that competitively binds to RANKL, thereby inhibiting the interaction between RANKL and RANK on the OC surface and suppressing OC differentiation and maturation (Ye, 2014). A concentration of 0.05 mg/L CAT significantly enhances the expression of OB OPG mRNA in the OB-OC co-culture system (Lai et al., 2016), mediating the process involved in regulating the expression of RANKL. Furthermore, CAT mitigates the ubiquitination and degradation of phosphatase and tensin homolog (PTEN), upregulating its activity. This leads to the inhibition of RANKL-induced phosphorylation and degradation of IκBα, subsequently suppressing the phosphorylation and nuclear translocation of NF-κB p65 (Meng et al., 2020). The downstream phosphorylation processes of AKT and GSK 3β are also inhibited. Additionally, CAT decreases the mRNA and protein expression levels of NFATc1, thereby suppressing the expression of its downstream genes including tartrate-resistant acid phosphatase (TRAP), cathepsin K (CTSK), dendritic cell-specific transmembrane protein (DC-STAMP), calcitonin receptor (CTR), V-ATPase d2 (Udagawa et al., 2021), and Oscar (Tokunaga et al., 2020). During the initial phases of osteoclastogenesis, CAT demonstrates the ability to suppress F-actin assembly in osteoclasts via RANKL-mediated pathways. thereby suppressing bone resorptive activity. This suggests that the role of CAT in inhibiting excessive osteoclast activity may be attributed to blocking the RANKL/RANK signaling pathway or targeting the AKT/GSK 3β/NFATc1 signaling cascade (Meng, 2020), mitigating the adverse impacts of inflammation and ovariectomy on bone density in mouse experimental systems (Udagawa et al., 2021). The data underscore CAT’s contribution to the synchronized modulation of bone turnover, indicating its potential as an innovative pharmacological intervention for osteoclast-mediated skeletal disorders.

### 3.2 Sirt 6/ERα/FasL signaling pathway

Through GO and KEGG pathway enrichment analysis, it has been found that the main components of processed Rehmannia glutinosa in treating OP may be related to the estrogen signaling pathway, HIF-1 signaling pathway, among others (Xie, 2022). Estrogen induces apoptosis of mature osteoclasts by activating the Fas/Fas ligand (Fas) pathway through the estrogen receptor (ER) α (Hu et al., 2023), participates in bone metabolism, and maintains bone formation (Khosla et al., 2012). Both *in vivo* and *in vitro*, CAT upregulates the expression of osteoclast apoptosis-related proteins including FasL, cleaved-caspase 8, cleaved-caspase 3, and Bax (Nakamura et al., 2007), indicating that CAT induces osteoclast apoptosis, reduces osteoclast numbers, and consequently decreases bone resorption. CAT increases the protein levels of deacetylases and nucleotidyl transferase Sirt 6, inducing deacetylation of ERα, and significantly upregulating ERα protein expression during the apoptosis process. This effect further influences the mRNA levels of FasL related to osteoclast apoptosis through ERα transcriptional activity, ultimately activating osteoclast apoptosis (Chen et al., 2024). The interactions within this complex regulatory network underlie the mechanism regulating osteoclast apoptosis. Consequently, the induction of osteoclast programmed cell death via the SIRT6-ERα-FasL signaling cascade may mitigate estrogen deficiency-induced osteoporosis.

## 4 Other anti-OP pathways

### 4.1 Antioxidant stress damage

The beneficial effects of CAT on osteoblast protection are primarily associated with its antioxidant activity (Zhu et al., 2016). CAT facilitates bone mineralization and mitigates the progression of OP through anti-inflammatory treatment strategies and by mediating the homeostasis of reactive oxygen species, reducing pain and improving bone damage in patients (Conaghan et al., 2019). CAT can attenuate impaired osteogenic differentiation, enhance the expression of COL1, BMP9, and RUNX2 (Zheng et al., 2020), and induce the phosphorylation of GSK3b (Eiraku et al., 2019). It reverses the reduction of serum GSH and SOD as well as the increase of serum MDA (Xu et al., 2023). Concentrations of 0.01–1 μM can inhibit the autophagy upregulation-induced OB apoptosis by 2,3,7,8-tetrachlorodibenzo-p-dioxin (TCDD), offering protection against oxidative stress. CAT restores the expression of cytoplasmic Cu/Zn SOD and increases the expression of the apoptosis regulatory factor GPx 4 gene in damaged cells (Choi et al., 2019), causing OBs to adapt to oxidative stress conditions. It also inhibits the elevation of phosphorylated ERK, consequently attenuating the elevated production of NO and inflammatory mediators (Ajizian et al., 1999), or by directly blocking the formation of NO/nitrites to alleviate nitrative stress, lowering the impact of oxidative stress through pathways such as the reduction of cytochrome P450 superfamily enzyme CYP1A1 (Choi et al., 2019), inhibiting bone loss. By activating the PKD 1/Sirt 1 pathway, it resists oxidative stress (Xu et al., 2023), and cooperatively enhances both ALP functionality and RUNX2 levels in BMSCs under oxidative stress (Chen et al., 2021), promoting proliferation and osteogenic differentiation.

### 4.2 Bone repair functions

CAT significantly promotes the migration and bone formation of MSCs, enhances the osteogenic activity of MSCs, enhances the osteogenic potential of MSCs in repairing rat osseous lesions, and increases bone density, making it useful for local bone repair. Thus, it facilitates the filling of bone defects in mice, maintains the chondrocyte phenotype, improves the fixation of bone structure and matrix, and effectively treats local bone defects (Wu et al., 2024). Previous studies have reported that experimental findings in rats reveal CAT’s capacity to potentiate BMSC-driven bone repair in substantial cranial defects and counteract post-ovariectomy osseous tissue loss (Zhu et al., 2019). Additionally, Research indicates that CAT suppresses TNF-α and COX-2 expression, mitigates nicotine-induced alveolar bone deterioration, and enhances osseous mineralization (Li et al., 2017a), suggesting its potential as a safe and efficacious intervention for bone loss.

### 4.3 Anti-oxidative and glucose deprivation damage

The anti-OP effect of Catalpol is also reflected in the protection of bone cells. Pre-treatment with CAT can enhance the survival and activity of BMSCs following oxygen-glucose deprivation (OGD) treatment. This phenomenon is characterized by elevated expression of the anti-apoptotic factor Bcl-2, coupled with reduced levels of pro-apoptotic Bcl-2 family members, including Bax, and the key mediator of apoptosis, Caspase-3. *In vivo* experiments have demonstrated that CAT can improve the survival rate of transplanted BMSCs (Ju et al., 2018).

### 4.4 Immune cell polarization

The pathogenesis of OP, a widespread inflammatory bone disorder, is intricately linked to immune system function (Srivastava et al., 2018). Th 1/Th 2 cells are important immune cells, having key functions in maintaining immune activation and immune tolerance. Th 1 cells produce inflammatory cytokines such as IL-12 and IFN-γ, which are associated with immune suppression and can strongly promote the formation of OC. Conversely, Th 2 cells secrete IL-4 and IL-10, which have opposite effects. This indicates the potential role of Th 1/Th 2 balance in bone remodeling and OP (Fujii et al., 2012). CAT intervention has been shown to effectively counteract bone depletion associated with estrogen insufficiency by favoring Th2 over Th1 in the immune profile, thereby altering the ratio of inflammatory cytokine production and mitigating OP caused by bone loss.

Macrophages exhibit plasticity, adopting either a pro-inflammatory M1 profile or an anti-inflammatory, tissue-restorative M2 configuration (McWhorter et al., 2013). Macrophage functional dichotomy in fracture healing involves M1-driven acute inflammation and site cleaning, followed by M2-mediated support of MSC osteogenic activity through secreted growth factors (Murray and Wynn, 2011). CAT can modulate the polarization phenotype of macrophages, reducing inflammation, decreasing the expression of M1-related factors, and, through interactions with osteogenic and other related cells, effectively promote osteogenesis, angiogenesis, and inhibit osteoclast production (Zhang et al., 2022). This suggests that CAT could enhance bone tissue regeneration and functional reconstruction through immune regulation (Wu et al., 2022).

### 4.5 Osteogenesis-angiogenesis coupling

**FIGURE 4:**
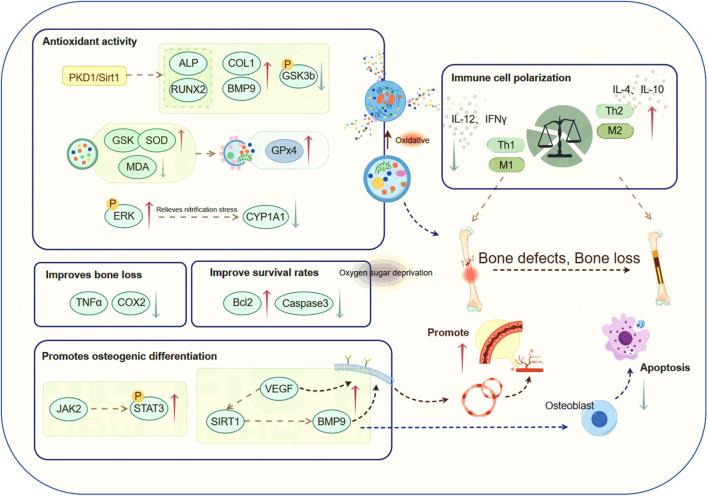
CAT has anti-osteoporotic effects through other pathways. Among them, CAT is involved in the mechanism research of regulating the course of osteoporosis through various pathways such as antioxidative stress injury, bone repair, antioxidant glucose deprivation injury, regulation of immune cell polarization, and promotion of osteogenesis-angiogenesis coupling.

Bone formation and bone healing are closely associated with angiogenesis (Kan et al., 2022). The osteogenesis-angiogenesis coupling process can be enhanced by various regulatory factors (Zou et al., 2023). Among them, the JAK/STAT signaling pathway is an indispensable and critical signaling pathway in the bone regeneration process. CAT can promote the activation and phosphorylation of STAT 3 by JAK 2. This leads to the dimerization and nuclear translocation of STAT 3, promoting osteogenic differentiation and enhancing the healing of bone defects (Wang and Xue, 2018), as well as stimulating the expression of the angiogenic factor VEGF in bone marrow multipotent progenitor cells and BMSCs (Wang et al., 2007; Liu et al., 2009). It participates in the recruitment, proliferation, and differentiation of BMSCs and vascular endothelial cells. Additionally, through the activation of SIRT 1, it promotes angiogenesis and osteogenic differentiation induced by BMP 9 in MSCs (Lu et al., 2022). CAT also facilitates angiogenesis and osteogenic activity is augmented by paracrine factors governing mesenchymal stromal cell-macrophage interactions (Zhang et al., 2022), thereby accelerating the repair of osteoporotic bones.This work forms a cornerstone for the clinical deployment of CAT in osteoporotic fracture management (Figure 4).

## 5 CAT future directions and recommendations in OP research and treatment

To further explore the potential of CAT in the treatment of OP, we propose the following recommendations and future research directions.

### 5.1 In-depth mechanism research

Although various studies have demonstrated the alleviating and ameliorating effect of CAT on OP, its exact molecular mechanism has not been fully elucidated. Conducting in-depth mechanistic studies will help to understand the exact site of action of CAT in the treatment of OP and provide more comprehensive evidence for its clinical application. Patients with OP often experience impairment of multiple systems. It is also crucial to identify common treatment pathways for the prevention and treatment of these diseases. Not only will it be possible to discover deeper molecular mechanisms, but it may also be possible to discover other synergistic protective effects of OP.

### 5.2 Effectiveness and toxicity studies

The effectiveness of a drug is largely related to the dosage of the drug used and depends on the route of administration. Therefore, we summarized the dosage and mechanism of action of CAT used in previous studies (Table 1). CAT is well tolerated and non-toxic. There are few clinical studies involving the relieving therapeutic effect of CAT on OP. To bridge the gap between preclinical and clinical studies and bring CAT into clinical use as soon as possible, well-designed clinical trials should be conducted to evaluate the efficacy, safety, and optimal dose of CAT in human subjects. It also provides a variety of protective effects according to the unique needs of different patient groups, and the individualized medication approach can be used to optimize the use of CAT in OP considering the individual differences in treatment response. By considering the individual patient’s genetic background, lifestyle factors, and disease progression, customized treatment strategies can be developed to maximize the therapeutic benefits of catalpa alcohol. In addition, further toxicity studies should be conducted to determine the long-term safety profile of CAT and its side effects.

Although there are currently no clinical studies on the efficacy of CAT in the treatment of OP, CAT has been used clinically to treat cardiovascular diseases, tumors, and other conditions. In clinical applications, Rehmannia has achieved good feedback and results in the treatment of OP (Li et al., 2022). We believe that CAT, as the main active ingredient of Rehmannia, has a very broad clinical prospect for the treatment of OP, potentially providing a new drug treatment option for OP patients. At the same time, in-depth research on CAT has opened a new avenue for developing and utilizing various natural medicinal components in treating clinical diseases.

## 6 Summary and outlook

CAT holds broad medicinal value and application prospects due to its effects on multiple organ tissues. Its multi-target action in reducing bone resorption, enhancing bone formation, and decreasing bone loss has garnered widespread attention in OP treatment (Zhang et al., 2016). CAT typically exerts its regulatory effects on bone metabolism through various molecular mechanisms such as estrogen-like activity, antioxidant activity, or regulation through multiple signaling pathways. These effects are pronounced, presenting significant potential and market value in drug and health supplement development. Research findings indicate that in Ovx mice treated with CAT, there was no significant increase in serum estradiol (E2) levels, the findings indicate that CAT may serve as a secure treatment option for mitigating estrogen deficiency-induced bone deterioration, avoiding the adverse effects typically linked to estrogen supplementation (Lai et al., 2015). Additionally, by utilizing CAT’s ability to promote osteoblast mineralization in conjunction with orthopedic implants, achieving effective local concentrations significantly improved osteogenesis. Thus, CAT loading enhances the bone integration effects of composite materials relative to conventional materials (Luyi, 2021), offering improvements to current therapeutic methods.

However, the mechanisms of CAT in bone metabolic diseases remain an area for further study. The utilization of bioinformatics tools, *in silico* modeling, and molecular docking techniques enables researchers to explore the molecular underpinnings and specific targets of CAT in OP treatment, laying the groundwork for subsequent experimental research. CAT is widely available and can be extracted using various methods tailored to different materials to maximize yield (Zhang and Liu, 2019). Yet, CAT has not been applied in clinical practice. Prior to making clinical application decisions, researchers need to conduct further studies to introduce CAT into clinical practice, contributing to human health.
